# Efficacy and safety of long-term transcutaneous electroacupuncture versus sham transcutaneous electroacupuncture for delayed gastric emptying after distal gastrectomy: study protocol for a randomized, patient-assessor blinded, controlled trial

**DOI:** 10.1186/s13063-022-06108-z

**Published:** 2022-03-03

**Authors:** Kai-Bo Chen, Zhi-Wei Wu, Jun Wang, Ling-Hua Zhu, Xiao-Li Jin, Guo-Feng Chen, Mu-Xing Kang, Yi Huang, Hang Zhang, Le-Le Lin, Di-Ke Shi, Dan Wu, Jian-Feng Chen, Jian Chen, Zhi-Qing Zhao

**Affiliations:** 1 Department of Gastrointestinal Surgery, The Second Affiliated Hospital of Zhejiang University (SAHZU), School of Medicine, No.88 Jie-Fang Road, Hangzhou, 310009 China; 2 Department of Gastrointestinal Surgery, Sir Run Run Shaw Hospital, Zhejiang University, School of Medicine, No. 3 East Qing-Chun Road, Hangzhou, 310020 China; 3 Department of General Surgery, Shang-Yu branch of SAHZU, School of Medicine, No. 517 Shi-Min Road, Shaoxing, 312300 China

**Keywords:** Transcutaneous electroacupuncture, Delayed gastric emptying, Distal gastrectomy, Gastric cancer

## Abstract

**Background:**

Delayed gastric emptying (DGE) after distal gastrectomy impacts patients’ nutritional status and quality of life. The current treatments of DGE seem unsatisfactory or need invasive interventions. It is unknown whether transcutaneous electroacupuncture (TEA) is effective in treating DGE.

**Methods:**

A total of 90 eligible participants who underwent distal gastrectomy will be randomly allocated to either the TEA group (*n* = 60) or the sham transcutaneous electroacupuncture (sham-TEA) group (*n* = 30). Each participant will receive TEA on the bilateral acupoints of Zusanli (ST36) and Neiguan (PC6) for 4 weeks. The primary outcomes will be the residual rates of radioactivity in the stomach by gastric scintigraphy and total response rates. The secondary outcomes will be endoscopic features, autonomic function, nutritional and psychological status, serum examination, and quality of life (QoL). The adverse events will also be reported. The patients will be followed up 1 year after the treatment.

**Discussion:**

The findings of this randomized trial will provide high-quality evidence regarding the efficacy and safety of long-term TEA for treating DGE after distal gastrectomy.

**Trial registration:**

Chinese Clinical Trial Registry ChiCTR2000033965. Registered on 20 June 2020

## Background

Gastric cancer is a common and lethal malignant disease worldwide. Although mortality has decreased substantially, approximately 0.5 million patients die of gastric cancer in China annually [1, 2]. Early diagnosis of gastric cancer and radical gastrectomy improves the prognosis. However, patients have the possibility of rapid or delayed gastric emptying after distal gastrectomy, which impacts their nutritional status and quality of life (QoL) [3, 4]. Specifically, 5–30% of patients develop delayed gastric emptying (DGE) following gastrectomy [5–7]. Postsurgical gastroparesis syndrome (PGS) is also used for this complication [8]. Tomita et al. found that epigastric fullness occurred in 60% of patients who had received pylorus-preserving distal gastrectomy 5 years ago, gastric stasis in the remnant stomach occurred in 40% of these patients, and the gastric emptying function of a liquid and semisolid diet was decreased [9]. Kim et al. showed that over half of postoperative gastric cancer patients had DGE with frequently observed gastric stasis and reflux esophagitis [10]. Jung et al. reported that the incidence of food retention was 55.5%, 31.9%, and 20.9% at 3, 12, and 24 months, respectively, after distal gastrectomy for early gastric cancer patients [11].

Nutritional support, glycemic control, prokinetics, antiemetic medications, endoscopic self-expandable stent placement, and surgical intervention in severe cases are considered to be helpful for DGE patients [12]. Nevertheless, prokinetics lack clinical evidence with possible severe side effects, and secondary surgery is not a preferred choice for postoperative patients.

Acupuncture is widely applied for gastrointestinal motility disorders, and it has been proven effective in DGE in critically ill patients and postoperative gastroparesis syndrome [13]. A meta-analysis suggested that acupuncture with or without medication exhibited a significantly higher total effective rate than control acupuncture [14]. Transcutaneous electroacupuncture (TEA) is modified from traditional acupuncture, and it works on specific acupoints combined with a skin electrode patch instead of acupuncture [15]. In animal models, electroacupuncture (EA) can improve gastric motility, accelerate solid gastric emptying via the vagal mechanism, and ameliorate dyspepsia induced by chemotherapy [16]. A small number of clinical studies proved that TEA could significantly improve gastric emptying in dyspeptic patients [17]. Our previous randomized, controlled, single-center study demonstrated that TEA improved bowel movement and alleviated postoperative ileus after gastrectomy [18].

In this study, we will compare long-term TEA versus sham transcutaneous electroacupuncture (sham-TEA) for DGE after distal gastrectomy. To the best of our knowledge, this is the first randomized, patient-assessor blinded, controlled trial on this subject.

## Methods/design

### Objectives and hypothesis

This study aims to (1) assess the efficacy and safety of long-term TEA for treating DGE after distal gastrectomy and (2) evaluate postgastrectomy patients by endoscopic features, autonomic function, nutritional and psychological status, serum examination, quality of life, and TEA-related adverse events. We hypothesize that TEA can safely improve DGE after distal gastrectomy.

### Study design

**Fig. 1:**
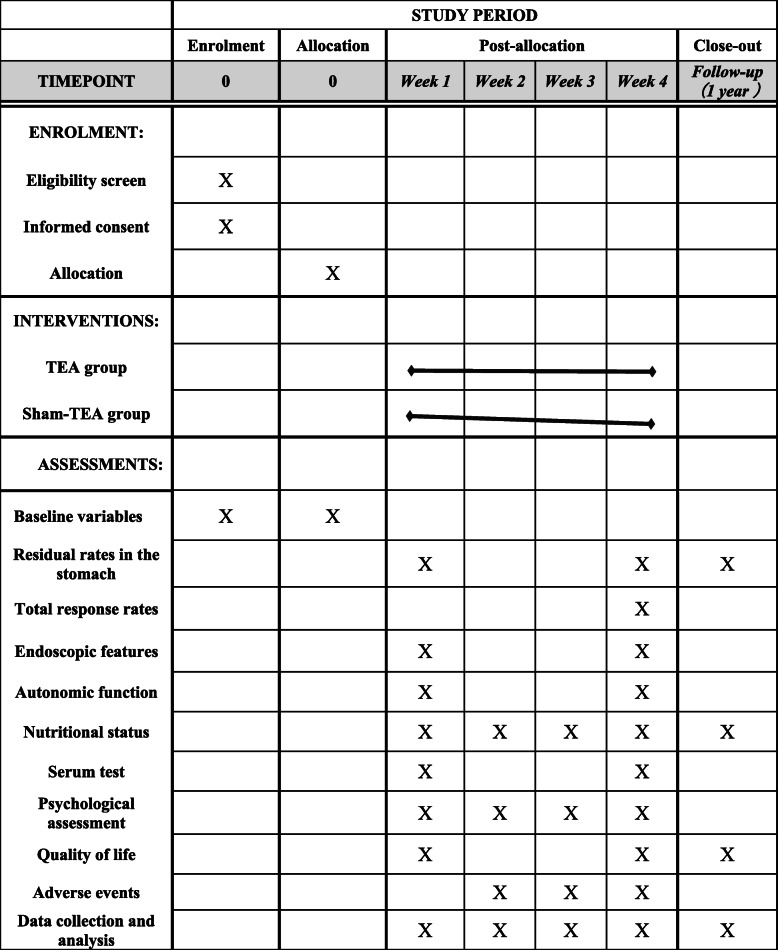
SPIRIT schedule of enrollment, interventions, and assessments

The trial will be conducted in the Second Affiliated Hospital of Zhejiang University, School of Medicine. This protocol was approved and monitored by the Medical Ethics Committees of the Second Affiliated Hospital of Zhejiang University, School of Medicine (approval number 2020-454), and Chinese Clinical Trial Registry (approval number ChiCTR2000033965) and will conform to the Declaration of Helsinki, STRICTA and SPIRIT guidelines (Fig. 1). The protocol of this study has been registered in the Chinese Clinical Trial Registry with the number ChiCTR2000033965. All eligible participants will be required to sign an informed consent form before allocation.

### Inclusion criteria

All allocated patients met the following inclusion criteria: (1) 18–80 years old, (2) gastric cancer patients considered to have DGE (a half-life gastric emptying by gastric scintigraphy greater than 70 min), (3) within 3–6 months after distal gastrectomy, (4) no gastrointestinal obstruction before surgery, and (5) TNM staging < IV, no distant metastasis.

### Exclusion criteria

Patients who met any of the following criteria were excluded: (1) anastomotic stricture or afferent loop obstruction of the jejunum diagnosed by gastroesophageal radiography, (2) taking drugs such as prokinetics or antidepressant drugs a month prior to surgery or during the trial, (3) severe arrhythmia, (4) tumor recurs during the trial, (5) diabetes mellitus, or (6) mental and physiological illness.

### Dropout criteria

Patients who choose to quit the study voluntarily or have poor compliance during the trial will be considered as having dropped out. Patients can discontinue the trial if a severe adverse effect occurs, and they can adjust the parameters of TEA slightly if the electric current makes them feel uncomfortable during the TEA procedure.

### Recruitment

**Fig. 2:**
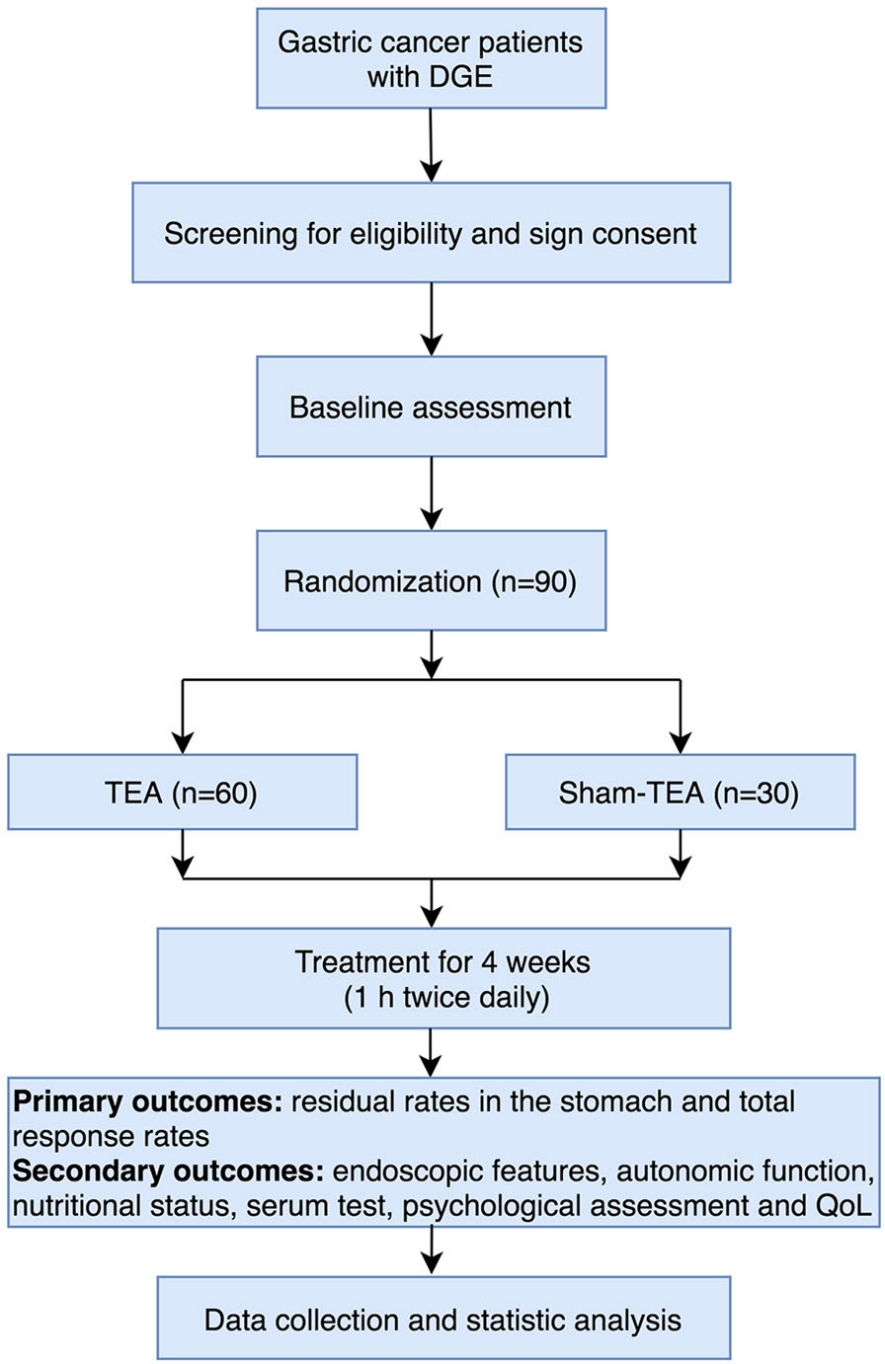
Flow diagram of the study

A total of 90 gastric cancer patients who underwent distal gastrectomy at the gastrointestinal surgery department of the Second Affiliated Hospital of Zhejiang University, School of Medicine, will be included in this study (Fig. 2). Demographic information of the patients will be collected.

All patients will undergo laparoscopic exploration with peritoneal lavage cytology to exclude peritoneal metastasis. The patients will undergo distal gastrectomy for the primary tumor, D2 or D1+ lymph node dissection with truncal vagotomy by a single clinical team. Billroth-I, Billroth-II, or Roux-en-Y reconstruction will be conducted using staplers and suture reinforcement, and Braun anastomosis will not be performed for Billroth-II reconstruction. Patients who have a high risk of recurrence will receive adjuvant chemotherapy.

### Randomization

All eligible patients will be randomly assigned to the TEA group or the sham-TEA group by an allocation ratio of 2:1, and the framework of the trial is assumed as superiority (Fig. 2). The randomization process will be achieved by opening sealed envelopes with computer-generated random numbers by the SPSS 22.0 software. The process will be implemented by a researcher who is not involved in the data collection or analysis.

### Interventions

The researchers have been trained in the TEA technique in Pace Translation Medical Research Center, Ningbo, China, and they will teach the patients or their relatives how to implement TEA at home, with the help of a written or electronic user manual. Patients or their relatives can report adverse events by telephone or internet consultation.

#### TEA group

TEA will be performed with a skin electrode patch (50 × 50 mm) accurately placed on bilateral acupoints of Zusanli (ST36) and Neiguan (PC6) based on the theory of traditional Chinese medicine and our previous study [19]. The TEA group will receive TEA 1 h twice daily for 4 weeks, and the parameters of TEA have been well designed for each acupoint: 0.1 s on, 0.4 s off, 100 Hz, 0.25 ms, 2–6 mA at PC6, and 2 s on, 3 s off, 25 Hz, 0.5 ms, 2–6 mA at ST36. The patients will feel mild electric current intermittently but not over the pain threshold, and they are free to perform a slight activity.

#### Sham-TEA group

The sham-TEA group will be performed in the same way except that the acupoints are 15 cm away from accurate acupoints. Ultimately, they will be evaluated for clinical outcomes after interventions for 4 weeks.

### Blinding

The researchers who initially applied or taught the TEA or sham-TEA procedures did not participate in the statistical analysis. The patients and statisticians were blinded to the group assignments. If unblinding happens, the participants will be terminated from the trial immediately, and the data will be marked and not included in the final analysis. The researchers will record the reason in CRFs.

### Participant timeline

Enrollment and allocation will be conducted before the study. TEA or sham-TEA intervention will be given twice daily from week 1 to week 4. The patients’ gastric emptying function, nutritional status, and quality of life (QoL) will be assessed 1 year after the treatment as a follow-up. The schedule of enrollment, allocation, postallocation, and close-out is shown in Fig. 1.

### Outcome measures

#### Primary outcome measures

##### Gastric emptying function

We will evaluate the gastric emptying function of these postoperative patients who visit outpatient clinics. Gastric scintigraphy will help us to evaluate the gastric emptying function and has the advantages of being noninvasive and painless. ^99m^Tc tin colloid will be mixed with a semisolid diet of 200 g of rice gruel with a boiled egg. Radioactive signs will be recorded from diet intake to 120 min afterward, and the residual rates of radioactivity in the stomach at 45, 60, 75, 90, 105, and 120 min will be calculated by a half-life of 9 mTc. We will compare the recorded residual rates and the time to 50% residual rate between the two groups as the primary outcomes.

##### Total response rates

We will compare the total response rates between the two groups. Meanwhile, we will perform a subgroup analysis by different operation types of Billroth-I, Billroth-II, or Roux-en-Y reconstruction. In fact, some researchers reported that Roux-en-Y reconstruction could destroy the natural small-bowel pacemaker [20–22]. Subgroup analysis of laparoscopic and open surgery will also be included in our results.

#### Secondary outcome measures

##### Endoscopic features

DGE patients may have chronic inflammation of the upper digestive tract. Thus, endoscopy will be performed to determine reflux esophagitis, gastritis, and gastric stasis in the remnant stomach. We will compare the ratios of reflux esophagitis, gastritis, and gastric stasis between the two groups.

##### Autonomic function

An electrocardiogram (ECG) will be performed on the first day and the last day of this trial, and it will assess the sympathetic and parasympathetic activities using the spectral analysis of heart rate variability (HRV). We will calculate the ratios of the low-frequency (LF) band and high-frequency (HF) band of the HRV signal, which can assess the autonomic function of the patients. A few studies have shown that electroacupuncture improves clinical outcomes via vagal activation [23–25]. ECG can assist us in revealing the mechanism of the effects of TEA on DGE.

##### Nutritional status

The change in serum albumin and body mass index (BMI) will be recorded for nutritional status screening and evaluation. Additionally, the scored patient-generated subjective global assessment (PG-SGA) (weight, food intake, symptoms, activities and function, disease state, metabolic demand, and nutritional physical examination) will be used to quickly identify malnutrition status, and patients will be scored as A (well-nourished), B (moderately malnourished or suspected malnutrition), and C (severely malnourished).

##### Psychological status

Depression and anxiety symptoms will be assessed using the Hospital Anxiety Depression Scale (HADS). The HADS is a reliable self-rating scale questionnaire with seven items (scored from 0 to 3). A total HADS score over 10 indicates positive outcomes (moderate to severe symptoms).

##### Serum examination

The likely mechanisms of TEA are activation of somatic and peripheral nerves, anti-inflammatory effects, and functional preservation of the interstitial cells of Cajal [19]. Thus, blood will be taken for the analysis of serum norepinephrine (NE), tumor necrosis factor-α (TNF-α), interleukin-1β (IL-1β), interleukin-6 (IL-6), and glucose.

##### Quality of life (QoL)

The QoL of the patients after distal gastrectomy will be evaluated using the Short Form 36 (SF-36) questionnaire, including physical functioning, role limitations due to physical problems, bodily pain, general health perception, vitality, social functioning, role limitations due to emotional problems, and mental health. Higher scores indicate better QoL.

### Safety and adverse effects

The adverse events related to TEA or Sham-TEA will be monitored and reported by the patients themselves. It is possible to cause local events of TEA, such as skin bruising, itching, hematoma, pain, and muscular soreness, and systemic events, such as dizziness, arrhythmia, and gastrointestinal discomfort. Adverse effects will be recorded in the CRFs. The research team will provide costs of the treatment and corresponding financial compensation for these patients who suffer harm from trial participation according to the consent form.

### Sample size

Based on a previous electroacupuncture study on gastric emptying function, the response rate is approximately 50–70% [26–30]; however, the data of sham electroacupuncture are insufficient. Thus, we assumed that the response rates of the TEA group and sham-TEA group were 50% and 20%, respectively. Therefore, Δ is 30%. We estimated the sample size using the following formula:

$$ n=\frac{2{\left({\mu}_{\alpha }+{\mu}_{\beta}\right)}^2P\left(1-P\right)}{\Delta ^2} $$

A sample size of 66 patients will give us a power of 90% (*β* = 0.1) with a two-sided significance level of 0.05 (*α* = 0.05). To compensate for a 20% expulsion rate, a total of 90 patients will be required for the study. We will design a ratio of 2:1 (60 patients in the TEA group, 30 patients in the sham-TEA group) based on the response rates of the two groups.

### Data management and monitoring

The attending doctor who has performed the gastrectomy will be responsible for obtaining informed consent. Each participant will be required to sign an informed consent form and questionnaires and record personal information during the trial. The researchers will confirm the original data, collect all data on clinical outcomes very accurately, and store the data in the database safely. Clinical data will be carefully preserved using electronic and printed CRFs. To guarantee data quality, only outcome assessors will access the CRFs. Except for the researchers and the members of the ethics committee, people unrelated to the trial are not allowed to obtain access to CRFs. The personal information of the patients will not be disclosed in the public presentation. The data will only be used for this study. Biological specimens will be collected, stored, and analyzed in the current trial, and they are not intended for future use in ancillary studies. All data associated with the trial will be uploaded to the database of our hospital, and it will be monitored thoroughly by the Medical Ethics Committees.

### Statistical analysis

Sensitivity analysis will be performed to determine the impact of incomplete records on the results. Intent-to-treat analysis of the results will be performed if there is nonadherence or missing data. Only two data statisticians will have access to the final trial dataset, which will only contain coded data, and they will perform statistical analysis using the SPSS version 22.0 software. Student’s *t* test will be performed to assess the difference in the measurements (the residual rates of radioactivity in the stomach at 45, 60, 75, 90, 105, and 120 min) between the TEA group and the sham-TEA group. Paired Student’s *t* test will be used to compare the differences in serum albumin, NE, TNF-α, IL-1β, IL-6, glucose, LF/HF (sympathovagal ratio), and QoL at baseline and 4 weeks after intervention in each group. The *χ*^2^ test will be used for categorical data such as the PG-SGA, HADS, and adverse events. Univariate and multivariate analyses with multiple linear regression will be used to identify independent predictors of outcome measures. The results will be aggregated as median values. *P* values of < 0.05 will be considered as statistically significant.

### Quality control

To guarantee the quality of the study, all researchers were well trained in the TEA technique, and the patients or their relatives were taught to implement TEA at home, with the help of a written or electronic user manual. Pictures will be taken and uploaded to our database to check the proper acupoints and frequency of TEA each day. Plans to promote participant retention and complete follow-up are as follows: First, online video calls are available for consultation to resolve any technical issues of TEA implementation and to improve research adherence of the patients. Second, we will confirm the completion of TEA each week. Third, the expenses of round trips to the hospital and relevant examinations will be reimbursed to promote participant retention. Fourth, if the participants discontinue or deviate from the trial, we will collect as much data as possible for further analysis. Researchers will be fully apprised of the inclusion and exclusion criteria, and data will be documented in CRFs. A standard operating procedure will be consistently performed according to the study protocol. Drop-outs and their reasons will be recorded. The Medical Ethics Committees will audit the procedure of the trial, have access to these interim results and make the final decision to terminate the trial.

## Discussion

DGE occurs after upper gastrointestinal tract surgery, including gastrectomy, pancreatic surgery, and esophagectomy [5, 31–33]. As the prognosis of gastric cancer patients improves, it turns out to be the main concern for patients with DGE after distal gastrectomy. DGE impacts the patients’ nutritional status and QoL. Moreover, persistent DGE results in psychological diseases and even influences the prognosis of gastric cancer. However, the current treatments of DGE seem unsatisfactory or need invasive interventions, and prokinetics should be used cautiously due to their limited long-term efficacy and significant side effect. EA or TEA has been proven to be an effective treatment for improving gastrointestinal motility in either animal models or clinical cases [17, 23, 24, 34, 35]. Self-administrating TEA has been developed as a more convenient, patient-friendly treatment. It is promising for treating DGE after distal gastrectomy. The purpose of this trial was to evaluate the efficacy of TEA versus sham-TEA in improving gastric emptying function and the safety of TEA. The protocol of this trial is a well-designed, randomized, patient-assessor blinded procedure, which will make the results convincing. Notably, we compared the TEA group versus the sham-TEA group rather than the medical treatment or control group, which ruled out a placebo effect.

Gastric scintigraphy is the gold standard method for the diagnosis of gastroparesis and DGE, but there is no uniform approach due to the differences in the composition, volume, and calories of the meal; the protocol of monitoring; and the analysis of data during the examination [36]. The definition of DGE after gastrectomy is not well addressed or studied. We defined DGE as a half-life of gastric emptying greater than 70 min according to previous studies [9, 37]. Tougas et al. chose a low-fat meal consisting of ^99m^Tc-labeled and scrambled eggs, two slices of bread, strawberry jam (30 g), and water (120 ml); anterior and posterior images were taken at times of 1 min, 60 min, 120 min, and 240 min in all patients, and the distribution of percent gastric residual retention at different time points was analyzed in 123 volunteers [38]. Similarly, Tomita et al. mixed ^99m^Tc tin colloids with a semisolid diet of 200 g of rice gruel with a raw egg. A gastric emptying curve was drawn based on the residual rate of radioactivity in the stomach [9]. Kim et al. used ^99m^Tc tin colloid-raidolabeled boiled eggs, and DGE was defined if a half-time of emptying was over 72 min [37]. In our trial, such a radioisotope method with endoscopy will be used to evaluate the gastric emptying function of these postgastrectomy patients. We will have ^99m^Tc tin colloid mixed with a semisolid diet of 200 g of rice gruel with a boiled egg, which is more in line with the appetites of Chinese people.

Gastric dysfunction after distal gastrectomy may be associated with damage to a gastric pacemaker (located at the junction between the fundus and the body of the greater curvature) and truncal vagotomy [39, 40]. Some studies reported that Roux-en-Y reconstruction could result in Roux-en-Y stasis syndrome due to the destruction of natural small-bowel pacemakers [20–22]. Kim et al. found that DGE was associated with laparoscopic operation and duration of the postoperative period [37]. Chong et al. revealed that gastric emptying within a week after distal gastrectomy was markedly slower in Billroth-I than in Billroth-II, while gastric emptying at 3–6 months showed no difference between the two groups [41]. Accordingly, it is necessary for subgroup analysis of different types of operations.

Several studies have indicated that TEA could increase vagal activity and suppress sympathetic activity [23, 25, 42, 43]. The LF and HF bands of ECG and the LF/HF ratio can well reflect vagosympathetic activities. Thus, we will assess the sympathetic and parasympathetic activities using ECG.

There are several limitations to our study. First, this trial will be conducted in a single center, so selection bias may exist. We suppose that confounding factors of surgical techniques and postoperative management will be eased if distal gastrectomy is performed by a single medical team. Second, TEA or sham-TEA will be implemented by the patients themselves at home, so the quality of manipulations cannot be guaranteed. Third, symptoms of upper abdominal fullness, early satiety, nausea, and vomiting will not be recorded because most DGE patients present with no specific symptoms [11, 37].

## Trial status

Protocol version number V1, June 2020. The recruitment of this protocol is currently ongoing, and it is expected to be completed in December 2023.

## Data Availability

There have been no baseline or pilot data in this study protocol to date. The data will be securely stored during the trial, and the final results will be published in a peer-reviewed journal.

## Abbreviations

EA: Electroacupuncture
TEA: Transcutaneous electroacupuncture
sham-TEA: Sham transcutaneous electroacupuncture
DGE: Delayed gastric emptying
PGS: Postsurgical gastroparesis syndrome
ECG: Electrocardiogram
HRV: Heart rate variability
LF: Low-frequency
HF: High-frequency
BMI: Body mass index
PG-SGA: Scored patient-generated subjective global assessment
NE: Norepinephrine
TNF-α: Tumor necrosis factor-α
IL-1β: Interleukin-1β
IL-6: Interleukin-6
HADS: Hospital Anxiety Depression Scale
QoL: Quality of life
CRFs: Case report forms
